# Avoiding pitfalls when modelling ligands in macromolecular crystallography

**DOI:** 10.1107/S2059798326007047

**Published:** 2026-08-12

**Authors:** Oliver S. Smart, Andrew Sharff, Claus Flensburg, Clemens Vonrhein, Gérard Bricogne

**Affiliations:** a Global Phasing Limited 9 Journey Campus, Castle Park CambridgeCB3 0AX United Kingdom; Deutsches Elektronen-Synchrotron, Germany

**Keywords:** ligand validation, X-ray crystallography, structure-guided drug discovery

## Abstract

The determination of protein–ligand complex structures by X-ray crystallo­graphy is a cornerstone of modern structure-guided drug discovery. However, the process is complex and fraught with potential pitfalls at every stage, from data collection to final model deposition. The presence of flawed or misinterpreted ligand models in the Protein Data Bank (PDB) can misdirect scientific efforts that rely on them as the basis for new hypotheses and experiments. This article outlines a practical approach for ligand validation during structure determination. We discuss the application of validation tools, such as those in *Coot*, *MolProbity*, *Mogul* and *Buster-report*, to avoid errors. By re-examining several deposited PDB entries, we illustrate key pitfalls, including (i) modelling a ligand into absent or ambiguous electron density, (ii) incorrect chemical definitions (*e.g.* chirality, tautomers), (iii) poor fit of parts of a ligand to the electron density and (iv) data/model mismatches during deposition. We emphasize the importance of a ‘null-hypothesis’ approach and continuous critical assessment throughout the modelling process to improve the reliability of deposited structures.

## Abstract 2

Flawed protein–ligand X-ray structures can misdirect drug discovery based on PDB-deposited models. This review uses publicly available PDB examples to highlight potential pitfalls, from absent ligands to incorrect chemistry, and presents a practical validation approach to improve model reliability.

## 1. Introduction

Determining the structure of a macromolecular ligand-complex structure by X-ray crystallography is a complicated, multi-step process (Smart & Bricogne, 2015 ▸). Mistakes can occur at any step and affect the reliability of the final deposited ligand-complex structure, as noted in many previous studies (Davis *et al.*, 2003 ▸, 2008 ▸; Kleywegt, 2007 ▸; Joosten, Womack *et al.*, 2009 ▸; Liebeschuetz *et al.*, 2012 ▸; Cereto-Massagué *et al.*, 2013 ▸; Weichenberger *et al.*, 2013 ▸, 2017 ▸; Deller & Rupp, 2015 ▸; Smart & Bricogne, 2015 ▸; Adams *et al.*, 2016 ▸; Agirre, 2017 ▸; Wlodawer *et al.*, 2018 ▸; Feng *et al.*, 2021 ▸; Shao *et al.*, 2022 ▸; Casagrande *et al.*, 2025 ▸). Determining the structure of a ligand or fragment of interest is a key part of modern structure-guided drug discovery (Blundell, 2017 ▸; Ferreira & Andricopulo, 2017 ▸). It is therefore crucial to prevent the production of misleading macromolecule–ligand complex structures that would adversely affect subsequent studies based on such problematic models. The presentation *Validation of ligands: making decisions while modelling*, given at the CCP4 Study Weekend on 5th January 2024, and this paper review the pitfalls that can occur at each stage, using structures from the Worldwide Protein Data Bank (wwPDB; Berman *et al.*, 2003 ▸) to highlight cases of misinterpretations and how they can be corrected and redeposited. We aim to raise awareness of how easy it is to make mistakes, helping inexperienced crystallographers avoid such pitfalls in the first place.

## 2. Ligand-validation tools

A key tool for ligand modelling and validation is *Coot* (Emsley & Cowtan, 2004 ▸; Emsley *et al.*, 2010 ▸), which enables the interactive inspection of electron-density maps and easy-to-use interactive ligand fitting. *Coot* also provides essential tools for ligand validation (Emsley, 2017 ▸), including assessment of ligand distortions against a restraint dictionary. It can display *MolProbity* (Chen *et al.*, 2010 ▸) analysis of short steric contacts between the macromolecule and the modelled ligand, highlighting potential clashes (Emsley, 2017 ▸). Additionally, *Coot* features the *FLEV* tool, which produces a 2D schematic depiction of ligand–protein interactions, as pioneered by Wallace *et al.* (1995 ▸), and helps with checking the tautomeric form of the ligand of interest.

The *Mogul* tool (Bruno *et al.*, 2004 ▸), from the Cambridge Crystallographic Data Centre (CCDC), validates ligand geometry by analysing related small-molecule structures in the Cambridge Structural Database (CSD; Groom *et al.*, 2016 ▸). It compares bond lengths, bond angles, dihedral angles and ring conformations with those of similar groups in CSD structures. *Mogul* can be run interactively or from within the CCDC *Mercury* program (Macrae *et al.*, 2008 ▸). The *Buster-report* program (Smart & Bricogne, 2015 ▸) produces 2D schematic depictions of each compound of interest, annotated with the results of its *Mogul* analysis, highlighting unusual deviations from the current model relative to similar chemistry observed in the CSD. The wwPDB has adapted these 2D schematic depictions of *Mogul* results for use in the Validation Report service (Feng *et al.*, 2021 ▸).

To validate the geometry of fitted ligands, *Mogul* dihedral and ring conformation assessments are most useful, as they primarily probe soft degrees of freedom (Liebeschuetz *et al.*, 2012 ▸). *Mogul* bond-length and bond-angle comparisons are generally not particularly useful, as they assess stiff degrees of freedom that are dominated by the restraints applied to the bonds and angles (Liebeschuetz *et al.*, 2012 ▸). An analogy can be drawn to the assessment of molecular geometry in amino acids in proteins, where Ramachandran plots analyse the dihedral angles in the protein backbone (Ramachandran *et al.*, 1963 ▸). Similarly, amino-acid side-chain rotamer analysis examines dihedral angles to assess preferred conformations (Chen *et al.*, 2010 ▸; Hintze *et al.*, 2016 ▸) and is useful for model validation and improvement (Read *et al.*, 2011 ▸). Bond-length and bond-angle deviations are not useful as general validation metrics for proteins, although extreme outliers should be examined (Read *et al.*, 2011 ▸). *Buster-report* presents the *Mogul* bond-length and bond-angle results, but it would be better to display the deviations from the restraint dictionary target values instead. If *Grade*2 restraints (Smart *et al.*, 2021 ▸) are used, the target values will be based on *Mogul*; if restraints from another source are used, the *Mogul* bond and angle results will reflect a mixture of restraint target values and the deviations due to fitting.

In this paper we use some of the most generally applicable ligand-validation tools, but it should be noted that validation is not a ‘one size fits all’ process. We are not specifically considering the quality and validation of carbohydrate-containing proteins. The *Privateer* program is a crucial tool for validating structures containing carbohydrates and glycans (Agirre *et al.*, 2015 ▸; Dialpuri *et al.*, 2023 ▸, 2024 ▸). The procedures outlined here are also not applicable to structures from the *Pan-Dataset Density Analysis* (*PanDDA*) method (Pearce *et al.*, 2017 ▸), which analyses multiple related datasets to identify low-occupancy fragments. *PanDDA* structures should not be considered in isolation (Weiss *et al.*, 2022 ▸). Validation of metal-binding sites is another important topic (Zheng *et al.*, 2014 ▸) that is not covered here.

## 3. Checklist for ligand validation during model building and refinement

To avoid mistakes, it is crucial to apply ligand-validation tools at every stage of the structure determination. To support this process, we have compiled a detailed procedural checklist to guide researchers through the entire modelling process, which is available from the supporting information website https://gphl.gitlab.io/ligand-pitfalls-paper. The checklist has the following stages.

(A) Preparation of the initial model and difference map without the ligand of interest. Careful model building of the protein and solvent will help to enhance electron density resulting from ligand binding. A difference density map should be prepared and examined to evaluate whether the density obviously arises from ordered solvent, cryoprotectants and/or buffer molecules rather than from ligand binding.
(B) Preparation of the CIF restraint dictionary for the ligand of interest. This should be checked to determine whether the described molecule agrees with the known chemistry of the ligand of interest and is chemically plausible. As part of this, it is useful to inspect a 2D chemical diagram of the molecule, so the *Grade*2 (Smart *et al.*, 2021 ▸) restraint generator also produces one alongside the restraint dictionary. Loading the restraint dictionary into *Coot* allows inspection of the ‘ideal’ optimized geometry of the molecule and produces a 2D chemical diagram. For nonconfidential ligands, check whether the molecule is present in the PDB chemical component dictionary (Westbrook *et al.*, 2015 ▸) or the PubChem library (Kim *et al.*, 2024 ▸). *Grade*2 (Smart *et al.*, 2021 ▸) has options to perform these checks automatically.
(C) Fit the ligand of interest and refine the resulting protein–ligand complex model. Fitting can be performed interactively in *Coot* or with an automated tool. After refinement, ‘clean up’ the model by interactively fixing problematic parts of the protein structure, including modelled solvent molecules. *Coot*’s validation tools are useful for this, particularly the difference-map peak analysis. Several cycles of rebuilding followed by refinement may be necessary. Careful model building of the protein and solvent will help to make ligand electron density clearer.
(D) Model-build and refine a null-hypothesis structure (see Section 3.1 below).
(E) Validation of the ligand of interest from the refined structure compared with the null hypothesis. The checklist sets out eight questions whose investigation uses validation tools to examine the electron density, stereochemistry and non­bonded contacts of the modelled ligand of interest, compared with the null-hypothesis structure. To be credible, the proposed structure with the ligand of interest bound should outperform the null-hypothesis structure across the eight questions posed.
(F) Deposition to the wwPDB or an internal database. It is essential to ensure that the information deposited is correct for end-users of the structure to avoid problems.

The checklist is most appropriate for novel ligands and when the electron density for ligand placement is uncertain. It need not be applied exhaustively. The case studies discussed in the following section illustrate what can go wrong when the principles outlined in the checklist are overlooked.

### 3.1. Modelling a null-hypothesis structure

While forming a null hypothesis (Piedmont, 2023 ▸) and testing it is a central paradigm in many areas of science (Morey *et al.*, 2018 ▸), in protein crystallography researchers routinely produce a single final model without considering alternatives (Pozharski *et al.*, 2013 ▸). This is a dangerous approach as it is prone to confirmation bias, particularly when modelling ligands (Wlodawer *et al.*, 2018 ▸). One approach to avoid this is to adopt a null hypothesis that the ligand of interest has not bound and instead that the electron density is due to other molecule(s), for example the following.

(i) Ordered solvent molecules. It is useful to compare the putative ligand’s electron density with that of water molecules visible elsewhere in the structure and in the apo structure (if available). Examples from the PDB where ligands have been modelled into electron density that is better explained by ordered water molecules are PDB entries 3ib0 (Pozharski *et al.*, 2013 ▸; Smart & Bricogne, 2015 ▸), 1jq8 (Pozharski *et al.*, 2013 ▸; Wlodawer *et al.*, 2018 ▸) and 3hnb, as shown in Section 4.2 below.
(ii) Buffer molecules or cryoprotectants. For example, Wlodawer *et al.* (2018 ▸) show that PDB entry 3e3y has a glutamine ligand of interest built into electron density that is better explained as a HEPES buffer molecule.
(iii) Ions. PDB entry 2vcy is the crystal structure of 2-enoyl thioester reductase of human FAS II (Chen *et al.*, 2008 ▸). This structure was co-crystallized with the NADPH cofactor ligand of interest, but electron density at the binding site clearly showed that sulfate ions were present instead, and the structure was refined and deposited without the ligand of interest. Modelling techniques were used to produce a model of the enzyme with the cofactor (Chen *et al.*, 2008 ▸).
(iv) Endogenous ligands. These are natural ligands that have been copurified with the protein. PDB entry 8ivl is an example (see Section 4.2 below).

Rather than assuming that a blob of electron density must arise from the ligand of interest, we recommend producing an additional null-hypothesis model. For novel ligands, identify a combination of ordered solvent, ions, buffer molecules, cryoprotectants and endogenous ligands that best accounts for the observed density. A useful approach is to obtain restraint dictionaries for all molecules used as buffers, crystallization additives and cryoprotectants, and to fit the observed electron density and difference density in *Coot* using these molecules and water. For structures with related ligands, a reasonable null hypothesis may be that the parent compound has been soaked by mistake.

This should be a useful approach as it will give researchers confidence that their ligand-binding model is experimentally verified. A quotation from Richard Feynman is apposite:

The first principle is that you must not fool yourself – and you are the easiest person to fool. So you have to be very careful about that. After you’ve not fooled yourself, it’s easy not to fool other scientists.

(Feynman, 1974 ▸).

## 4. Case studies showing pitfalls when modelling ligands in macromolecular crystallography

We re-evaluate several PDB entries that illustrate issues in ligand modelling and refinement, as well as one positive control. A summary is provided in Table 1 ▸. A supporting information website (https://gphl.gitlab.io/ligand-pitfalls-paper) offers complete, step-by-step descriptions of the reinterpretations of each structure considered, including the commands executed, coordinate files, restraint dictionaries, electron-density maps, additional discussion and numerous figures.

### 4.1. A positive control

Before examining common pitfalls, it is instructive to review a well modelled structure. PDB entry 4tzt with ligand 468 (He *et al.*, 2006 ▸) serves as an excellent positive control.

Refinement with the actual ligand of interest 468 resulted in an excellent electron-density fit for the ligand (Supplementary Fig. S1-10), with a high real-space correlation coefficient (RSCC = 0.95), and passed all geometric and steric checks. Several null-hypothesis structures (Checklist stage D) were considered, including PEG 400 and the parent compound 566, in which the phenyl group replaces the chloromethylphenyl group (Supplementary Fig. S1-2). PEG 400 was considered because, although it was not listed in the crystallization conditions, it is a common cryoprotectant (Pflugrath, 2015 ▸) and might therefore be observed binding. The PEG 400 model fitted the electron density poorly (Supplementary Fig. S1-11;RSCC = 0.82) and resulted in significant restraint violations and steric clashes. The unsubstituted parent compound (566) model was a good overall fit and exhibited strong positive difference density peaks precisely where the additional chlorine and methyl groups in the ligand of interest (468) are located (Supplementary Figs. S1-9), thereby unambiguously confirming the identity and pose of the bound ligand. This case demonstrates that when properly applied, the checklist procedure robustly rejects incorrect alternative models and confirms the correct model.

### 4.2. Is there clear, unambiguous electron density to support the placement of the ligand?

Given an initial macromolecular structure with a soaked or co-crystallized ligand of interest, it is essential to carefully prepare electron-density maps to assess whether the ligand has bound and then use them to fit it. A standard approach is to refine and model-build the protein structure as completely as possible, without any modelled ligand. This model will hopefully produce a clear difference density map showing well defined features with the shape of the soaked ligand of interest. When calculating the difference map, it is important to ensure that the difference density is not distorted by either explicitly modelled water molecules or bulk-solvent masking in the putative binding-site region (Vonrhein & Bricogne, 2005 ▸; Vonrhein, 2011 ▸; Liebschner *et al.*, 2017 ▸).

Given a difference density blob, it is vital to critically assess whether it supports the placement of the ligand of interest. Could the density instead be caused by something else? PDB entries with a ligand of interest fitted into electron density that is better explained by other molecules are disappointingly common (Dauter *et al.*, 2014 ▸; Deller & Rupp, 2015 ▸; Smart & Bricogne, 2015 ▸; Wlodawer *et al.*, 2018 ▸). A helpful approach we demonstrate in this paper is to construct a null-hypothesis structure in which the optimal combination of ordered solvent, cryoprotectant or buffer molecules is used to account for the observed electron density at the presumed ligand-binding site. Following on from this, an alternative-hypothesis structure is modelled with the ligand of interest bound. To be credible, this alternative-hypothesis structure must fit the electron density better than the null-hypothesis structure and have no serious geometry validation issues. We will look at two cases in which structures have been deposited in the PDB with a ligand of interest despite a null hypothesis being better justified.

#### 4.2.1. Case study 1

**Figure 1:**
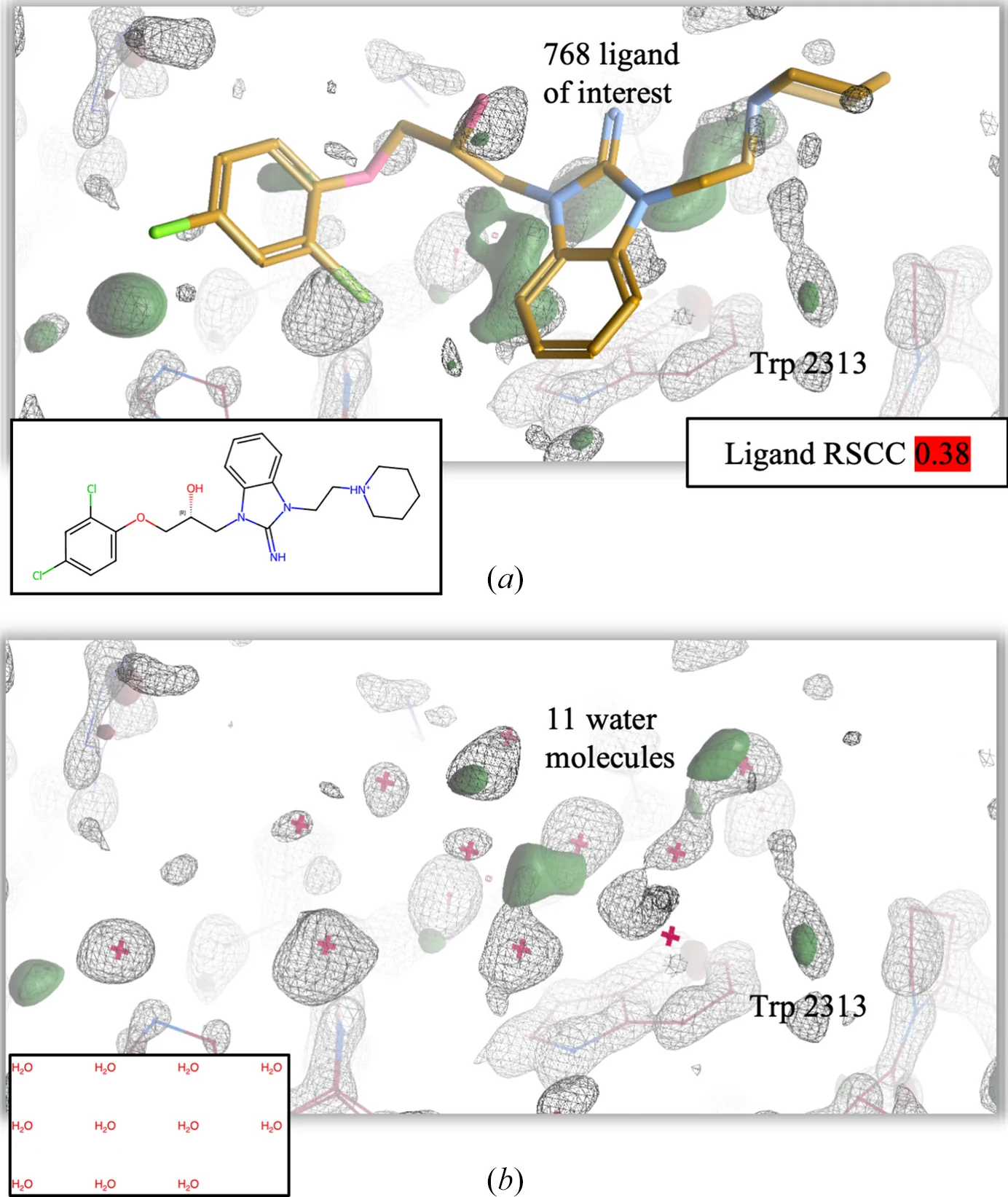
The inhibitor in PDB entry 3hnb has been fitted into electron density that is better explained by water molecules. (*a*) Re-solving the structure with the ligand of interest taken from PDB entry 3hnb shows that there is no electron density supporting the placement of the ligand. (*b*) The electron density in the site where the ligand was fitted is better explained by ordered water molecules. Figure produced using *Coot* with the *BUSTER* 2*mF*_o_ − *DF*_c_ map contoured at 1.3 r.m.s.d. shown as a grey mesh and the *mF*_o_ − *DF*_c_ difference maps contoured at 3.5 r.m.s.d. shown as red (negative) and green (positive) solid surfaces.

PDB entry 3hnb is a 1.14 Å resolution structure of the factor VIII segment, with a modelled inhibitor that blocks membrane binding (Liu *et al.*, 2010 ▸). Examination of the electron density around the modelled inhibitor in PDB entry 3hnb on the PDBe or RCSB websites, or using electron-density maps from *BUSTER* (Supplementary Figs. S2-1 and S2-4), shows no convincing density for the inhibitor. The RSCC for the inhibitor is well below 0.8, indicating a poor fit (Smart *et al.*, 2018 ▸). The *PDB-REDO* (Joosten *et al.*, 2014 ▸) re-refinement of PDB entry 3hnb confirms that this is a problematic ligand, with high *B* factors and only isolated patches of electron density nearby. Re-solving the structure, starting from the related entry 3hny (Liu *et al.*, 2010 ▸), which has no ligand bound, shows that an initial model, without the ligand of interest being placed, has no regions with difference density indicative of a ligand binding (supporting information website Section S2A). Examining the area where the inhibitor was modelled in PDB entry 3hnb shows that the site is occupied by a network of approximately 11 water molecules (Fig. 1 ▸). There are some patches of positive difference density near the water molecules. These could be due to alternative positions of the water molecules or to disordered ethylene glycol molecules (a crystallization additive). However, the small amount of difference density does not support the placement of a large ligand. In this case, the null hypothesis that the ligand of interest has not bound is evident from the start. Furthermore, comparing a re-refinement of the PDB entry 3hnb model with the null-hypothesis structure, in which water molecules occupy the site, yields better validation measures for the null-hypothesis structure (Supplementary Table S2A-1).

As the ligand of interest contains two Cl atoms, it is informative to compare its density with that of a chloride ion modelled in the reinterpreted structure (Supplementary Fig. S2A-11), which has contact distances and coordination number matching those expected for a chloride ion (Carugo, 2014 ▸). The chloride ion and S atoms in the methionine and disulfide produce clear r.m.s.d. > 10 density peaks (Supplementary Fig. S2A-13), whereas in PDB entry 3hnb there is no such density near the modelled inhibitor’s Cl atoms. The anomalous signal and specific radiation damage of halogen atoms can be exploited to confirm unambiguously binding positions for ligands containing them, even if they bind at low occupancy (Ma *et al.*, 2024 ▸; Rodrigues *et al.*, 2024 ▸).

#### 4.2.2. Case study 2

PDB entry 8ivl is a 2.70 Å resolution structure of fatty acid-binding protein 7 with cholesterol bound solved by Wei and coworkers (Fang *et al.*, 2024 ▸). As part of a study comparing many fatty acid-binding protein structures (Casagrande *et al.*, 2025 ▸; Ehler, Benz *et al.*, 2025 ▸; Ehler, Bartelmus *et al.*, 2025 ▸), Rudolph and coworkers have recently suggested that two PDB entries, 8ivl and 2qm9, contain ligands modelled into electron density which is better interpreted as co-purified endogenous fatty acids (Casagrande *et al.*, 2025 ▸).

**Figure 2:**
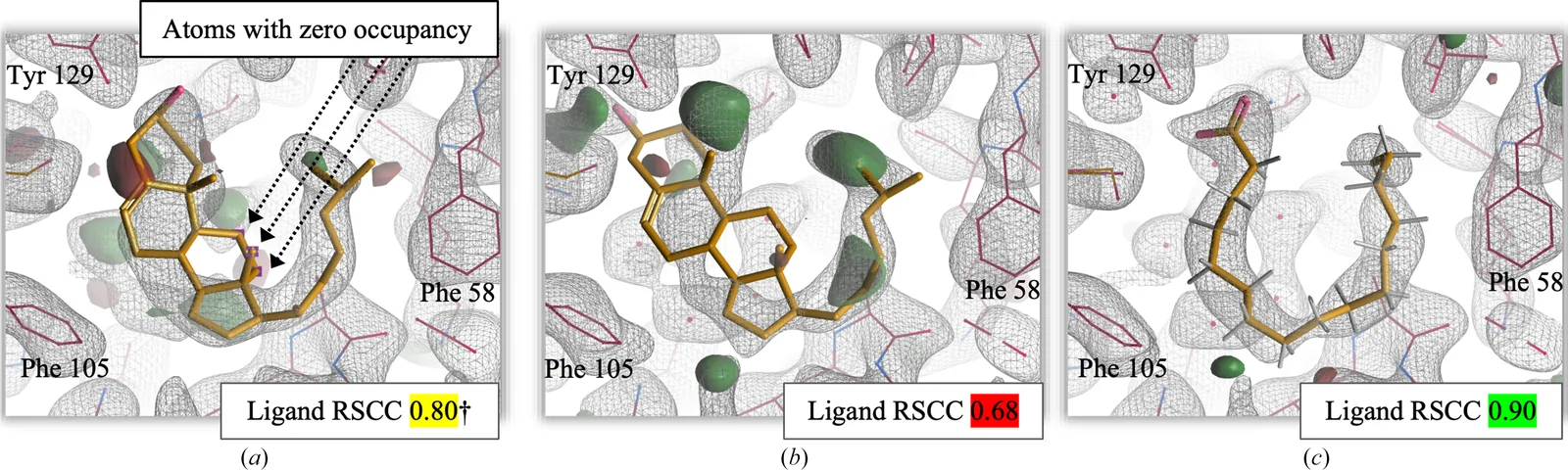
The cholesterol ligand of interest in PDB entry 8ivl has been fitted into electron density that is better explained by an endogenous fatty acid. (*a*) The cholesterol ligand in the PDB deposition is a poor fit to the electron density and has a strained conformation. † Note that the three atoms with zero occupancy do not contribute to the X-ray map calculation and consequently the RSCC is improved. (*b*) Refinement after *Coot* was used to interactively refit the cholesterol molecule to improve its geometry; this resulted in a worse fit to the electron density. (*c*) Modelling the ligand as the anion of a stearate fatty acid results in a much better density fit and in improved ligand-validation metrics. Figure produced using *Coot* with the *BUSTER* 2*mF*_o_ − *DF*_c_ map contoured at 0.8 r.m.s.d. shown as a grey mesh and the *mF*_o_ − *DF*_c_ difference maps contoured at 3.0 r.m.s.d. shown as red (negative) and green (positive) solid surfaces.

We have also re-examined PDB entry 8ivl; see Section S2B of the supporting information website. Our assessment is that after molecular replacement and an initial model cleanup and refinement, the electron density in the binding site does not resemble that of the ligand of interest, cholesterol. In PDB entry 8ivl, the cholesterol has been modelled with three atoms within the ring system having their atomic occupancy set to zero (Fig. 2 ▸*a*). In contrast, the other atoms in the cholesterol are modelled at full occupancy. As the cholesterol ring system is known to be rigid (Róg *et al.*, 2007 ▸), it makes no physical sense to model these three atoms as being entirely disordered. The cholesterol in PDB entry 8ivl (Fig. 2 ▸*a*) is conformationally strained, with the A ring and the adjacent hydroxyl group being bent into electron density. The bending causes internal short nonbonded contacts (Supplementary Fig. S2B-6) within the molecule and leads *Mogul* to assess that the A ring has a highly unusual bent-boat geometry compared with CSD structures (Supplementary Fig. S2B-7). *Coot* was used to interactively refit the cholesterol molecule so that the A ring has a reasonable geometry and to fix the atomic occupancy. *BUSTER* re-refinement of this model (Fig. 2 ▸*b*) results in the cholesterol adopting a more reasonable conformation but moving out of electron density and producing difference density. The RSCC for the cholesterol is 0.68, well below 0.8, indicating a poor fit. After the refit and re-refinement, the cholesterol molecule has restraint violations and internal short steric nonbonded contacts (Supplementary Fig. S2B-23). *MolProbity* analysis within *Coot* shows the ligand has four ‘bad overlaps’ with protein side chains (Supplementary Fig. S2B-25).

Assessing the difference density in the potential binding site after molecular replacement and re-refinement revealed that many peaks corresponded to bound water molecules (Supplementary Fig. S2B-13). In addition, there is a clear difference density blob (Supplementary Fig. S2B-14) for a larger molecule next to the side chains of Arg127 and Tyr129, which are known fatty acid-interacting residues (Casagrande *et al.*, 2025 ▸). A short fatty acid was built into the density and progressively extended following re-refinements (Supplementary Figs. S2B-16, S2B-17 and S2B-19). Refinement with the 18-carbon fatty-acid anion stearate produces a good fit to the electron density (Fig. 2 ▸*c*). The stearate has no validation metric issues, in contrast to the cholesterol ligand of interest (Supplementary Table S2B-1).

We concur with Casagrande *et al.* (2025 ▸) that the cholesterol modelled in PDB entry 8ivl has been placed into electron density that is due to endogenous fatty acid(s). The cholesterol in PDB entry 8ivl has a poor fit to electron density, even after three atoms have implausibly had their occupancy set to zero. In addition, the cholesterol in PDB entry 8ivl is conformationally strained and has bad contacts with the surrounding protein residues. We show that the electron density in the site can be well modelled as a stearate fatty acid, in agreement with Casagrande *et al.* (2025 ▸). Wei and coworkers (Fang *et al.*, 2024 ▸) appear to have fallen prey to an extreme case of confirmation bias.

These two examples highlight the critical importance of challenging the assumption that just because a crystal has been soaked with a ligand, the same ligand is present and visiblein the crystal structure. We recommend (Table 1 ▸) that the depositors of PDB entry 3hnb obsolete this entry without replacement to remove it from the main PDB, and that the depositors of PDB entry 8ivl replace this entry with the null-hypothesis structure in which stearate is modelled in place of cholesterol.

### 4.3. Is the chemical definition of the ligand in the restraint dictionary correct?

Modern ligand-restraint dictionary generators, such as *eLBOW* (Moriarty *et al.*, 2009 ▸), *Grade* (Smart *et al.*, 2010 ▸), *Grade*2 (Smart *et al.*, 2021 ▸) or *AceDRG* (Long *et al.*, 2017 ▸), will generally produce reasonable restraints for fitting and refinement when given the correct chemical definition of the ligand of interest. However, using a flawed chemical definition will yield an incorrect ligand-restraint dictionary and a poor model, even when the ligand is fitted into high-quality electron density.

#### 4.3.1. Case study

**Figure 3:**
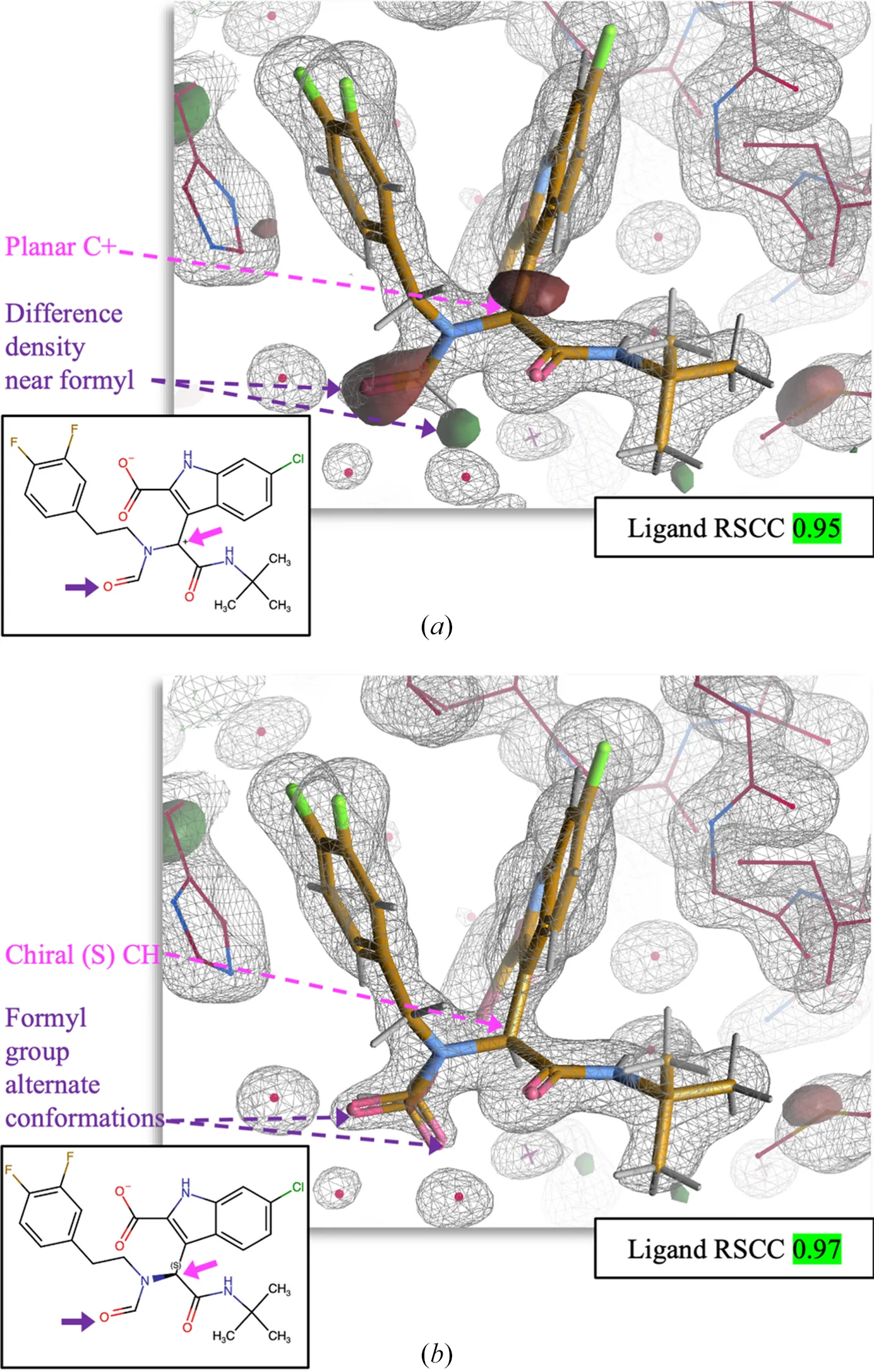
PDB entry 3tu1 shows the effect of a mistake in the chemistry of the ligand of interest. (*a*) In the original deposition, the ligand was modelled as a flat carbenium ion (indicated by a magenta arrow). Re-refinement of the PDB entry with *BUSTER* produces a strong negative difference density peak (red solid surface) near the ‘carbenium’ atom. There are additional difference density peaks near the ligand’s formyl group (marked with purple arrows). (*b*) Correcting the ligand’s chemistry to the (*S*) stereoisomer and modelling an alternative conformation of the formyl group eliminates the difference density peaks, resulting in a better overall fit. Figure produced using *Coot* with the *BUSTER* 2*mF*_o_ − *DF*_c_ map contoured at 1.3 r.m.s.d. shown as a grey mesh and the *mF*_o_ − *DF*_c_ difference maps contoured at 3.5 r.m.s.d. shown as red (negative) and green (positive) solid surfaces.

PDB entry 3tu1 is a 1.6 Å resolution structure of p53-MDM2 with a potent agonist (Huang *et al.*, 2012 ▸). The entry was modelled with ligand 07G, which was incorrectly defined as a planar C^+^ carbenium ion rather than a chiral CH group in the original PDB deposition (Supplementary Table S3-1 and Fig. S3-1). Re-refinement with restraints based on the incorrect chemical definition indicates that the ligand is reasonably well placed in the electron density (Fig. 3 ▸*a*) and has a real-space correlation coefficient above 0.95. However, there is a strong negative difference density peak near the C^+^ carbenium atom, indicating a poor local fit because this group has been forced to be planar rather than chiral (Fig. 3 ▸*a*). Correcting the chemical definition to the (*S*) stereoisomer and re-refining successfully eliminated the negative difference density at the chiral centre, leading to a better overall fit (Fig. 3 ▸*b*). The analysis also revealed and modelled previously ignored alternate conformations of the ligand’s formyl group, resulting in a significantly improved final structure. The preceding structure, PDB entry 3tj2 (S. Wolf, Y. Huang, G. M. Popowicz, S. Goda, T. A. Holak & A. Doemling, unpublished work), has a closely related ligand that was correctly modelled as an (*S*) stereoisomer (Supplementary Figs. S3-3 and S3-4).

This example shows that any difference density peaks near a ligand should be carefully considered. Furthermore, even PDB ligands with a high overall real-space correlation coefficient may still contain regions that are modelled incorrectly. We recommend that the depositors of PDB entry 3tu1 submit the reinterpreted structure as an update to the entry.

### 4.4. Part of the ligand has a poor geometry and/or fit

Even with correct chemistry and good electron density, parts of a ligand can be left in an unreasonable conformation.

#### 4.4.1. Case study

**Figure 4:**
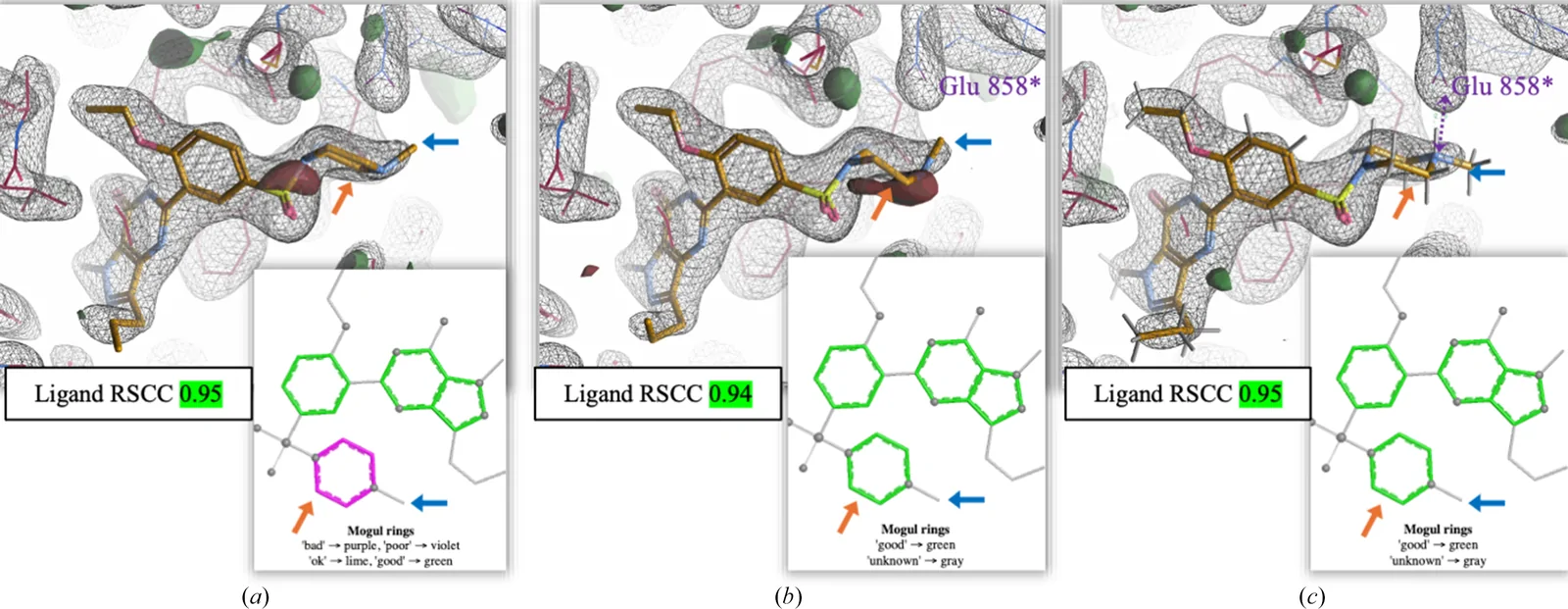
The sildenafil ligand in PDB entry 1udt is improved by refitting. (*a*) The sildenafil ligand of interest in PDB entry 1udt has a good fit to clear electron density, but the *N*-methylpiperazine ring was modelled with poor geometry. The piperazine ring (marked with an orange arrow) is not in a chair conformation, resulting in a poor *Mogul* ring score marked in purple in the *Buster-report* 2D graphic. Furthermore, the methyl group (highlighted with a blue arrow) is axial to the piperazine ring. (*b*) Re-refinement of the PDB entry with *BUSTER* improves the geometry of the piperazine ring so that the *Mogul* ring score is classified as ‘good’ by *Buster-report*. However, the methyl group remains trapped in an axial conformation. (*c*) Refitting sildenafil with the methyl group in an equatorial conformation yields a good fit to the electron density and favourable geometry. The piperazine is clearly protonated and forms a salt bridge with the side chain of a glutamate residue from an adjacent, symmetry-related protein molecule. The ligand’s overall fit to electron density, as assessed by RSCC, is as good as that in PDB entry 1udt, but refitting results in less difference density, better ring geometry and better ligand–protein contacts. Figure produced using *Coot* with the *BUSTER* 2*mF*_o_ − *DF*_c_ map contoured at 1.3 r.m.s.d. shown as a grey mesh and the *mF*_o_ − *DF*_c_ difference maps contoured at 3.5 r.m.s.d. shown as red (negative) and green (positive) solid surfaces.

PDB entry 1udt is a 2.3 Å resolution structure of human phosphodiesterase 5 (PDE5) complexed with sildenafil (Sung *et al.*, 2003 ▸). The sildenafil ligand of interest in PDB entry 1udt fits well in clear electron density (Fig. 4 ▸*a*) and shows a high real-space correlation coefficient of 0.95 (supporting information website Section S4). However, *Mogul* geometry analysis shows that the piperazine ring in the ligand has an unusual conformation (Fig. 4 ▸*a* and Supplementary Fig. S4-4). Probing the ring geometry using Cremer–Pople analysis (Cremer & Pople, 1975 ▸) shows that the piperazine is in a high-energy envelope conformation (Supplementary Fig. S4-5), rather than adopting the canonical chair conformation (supporting information website Section S4). Furthermore, the methyl group attached to the piperazine ring is in an unfavoured axial position (Supplementary Fig. S4-5). Re-refinement of the PDB entry improves the geometry of the piperazine ring (Fig. 4 ▸*b*). Still, the methyl group remains trapped in an axial position, resulting in an unfavourable short contact to Glu858 from a symmetry-related copy (Supplementary Fig. S4-7) and a large patch of negative electron density (Fig. 4 ▸*b* and Supplementary Fig. S4-7). We solved the problem by interactively refitting the sildenafil ligand in *Coot*, moving the methyl group from the problematic axial position to the energetically stable equatorial position (Supplementary Fig. S4-8). Refinement following this correction dramatically improves the ligand’s fit to the electron density, eliminating the negative difference map peak (Fig. 4 ▸*c*).

This new, corrected fit revealed that the sildenafil ligand is protonated, allowing its piperazine nitrogen to form a strong salt bridge with a glutamate residue from an adjacent, symmetry-related protein molecule (Fig. 4 ▸*c* and Supplementary Fig. S4-11). This intermolecular salt bridge, a result of crystal packing, accounts for the difference in the piperazine conformation relative to other PDB entries of sildenafil bound to PDE5 (Supplementary Fig. S4-28), as discussed by Wang *et al.* (2006 ▸).

Over and above the correction of the ligand geometry, many improvements in the protein structure were made, including adding a missing disulfide bond between Cys677 and a symmetry-related copy of the same residue (Supplementary Figs. S4-12 and S4-13). The missing intramolecular disulfide bond in PDB entry 1udt is described in Wang *et al.* (2006 ▸), who link the dimerization to the low catalytic activity reported by Sung *et al.* (2003 ▸). The improvements to the protein model, in turn, enhanced the map quality around the ligand and enabled the placement of a bridging water molecule between the sildenafil and a neighbouring glutamine residue (Supplementary Fig. S4-21). Identifying bridging water molecules can be important in ligand design (Maurer & Oostenbrink, 2019 ▸). The final refined conformation of the sildenafil ligand still has some remaining unusual geometry features in the sulfonyl linker (Supplementary Figs. S4-22, S4-23, S4-24 and S4-25). These can be attributed to the salt bridge with Glu858* (Supplementary Fig. S4-25), which distorts the sulfonyl linker relative to other sildenafil-bound PDE5 structures (Supplementary Fig. S4-26).

This example shows that refitting of a ligand is sometimes necessary, as re-refinement can be trapped in local minima. It also demonstrates that minor improvements to a protein structure can enhance map quality (Rupp, 2010 ▸), thereby revealing features near the ligand of interest. The remaining ‘unusual’ validation metrics for the correctly placed sildenafil ligand are not due to errors but rather to physically induced strains within the molecule arising from the salt bridge. It is important not to adjust models to obtain clean validation reports; instead, it is sensible to check whether experimental data justify unusual features.

### 4.5. Is the modelled ligand in the appropriate charge state and tautomer?

As a final check for a modelled ligand with a good electron-density fit and good geometric validation scores, it is sensible to ask: does the refined modelled ligand have sensible ligand–protein contacts? *Coot* can assess hydrogen-bond complementarity between the ligand and surrounding groups; the *FLEV* 2D schematic plot (Emsley, 2017 ▸) is helpful for this. If the hydrogen bonds do not make sense, then consider whether a charged or tautomeric form of the ligand would. Bax *et al.* (2017 ▸) provide an excellent review of tautomers and describe how about 25% of drugs have more than one tautomeric form.

We have already seen an example of assigning the charge state in Section 4.4, where consideration of electrostatic interactions allows the sildenafil ligand to be modelled in the cationic form, forming a salt bridge with a glutamate side chain.

#### 4.5.1. Case study

**Figure 5:**
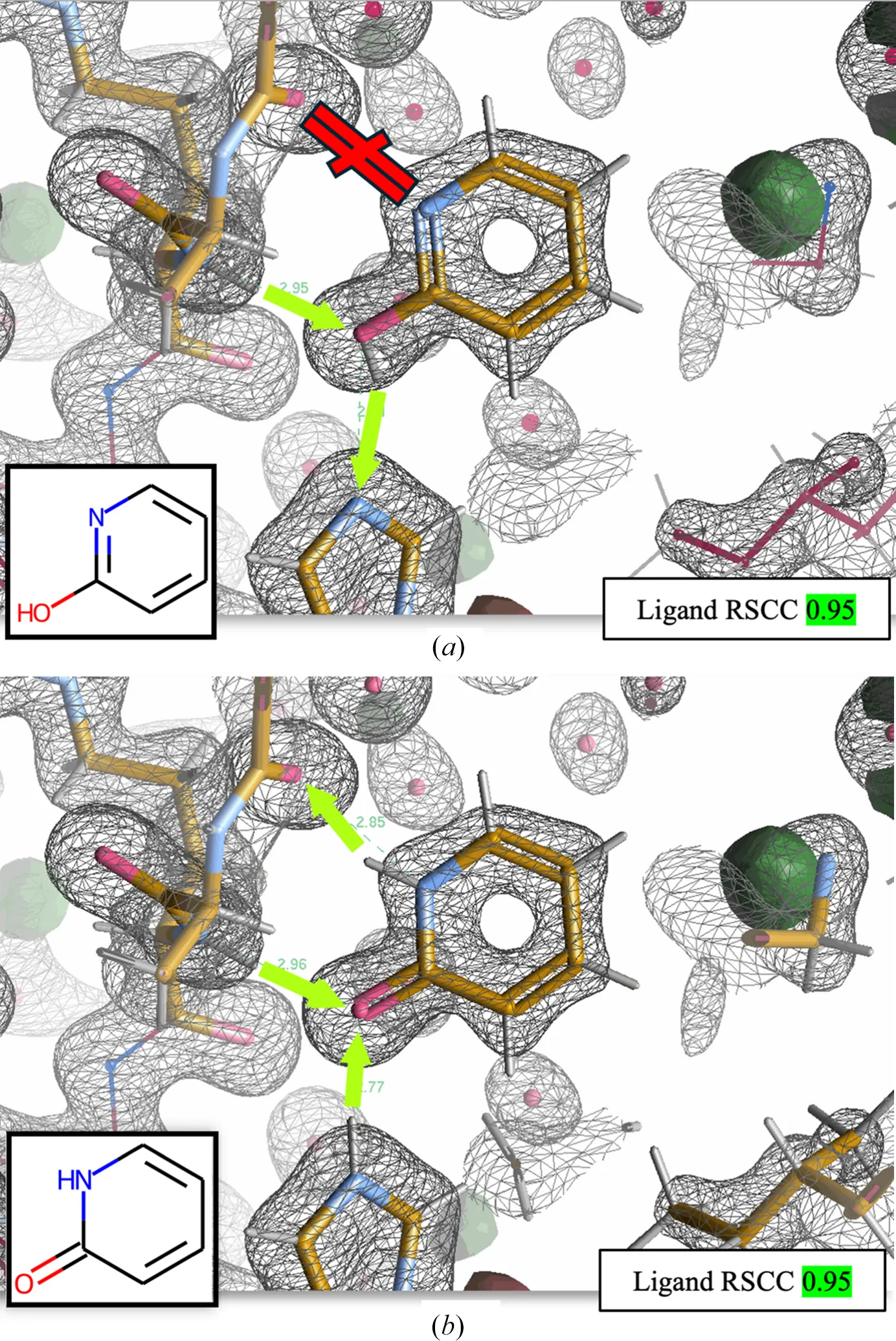
PDB entry 5s7c ligand A 509 shows how consideration of hydrogen-bond interactions can be used to assign the tautomeric state of a ligand. (*a*) The PDB entry models this ligand as 2-hydroxypyridine, which fits the electron density well and forms two favourable hydrogen bonds with the protein (marked by green arrows). However, the pyridine N atom points towards a main-chain carbonyl group, and the interaction between two hydrogen-bond acceptors is not favourable (marked by crossed red lines). (*b*) Remodelling the ligand to 2-pyridone yields an equally good fit to the electron density and three favourable hydrogen bonds (green arrows). Figure produced using *Coot* with the *BUSTER* 2*mF*_o_ − *DF*_c_ map contoured at 1.3 r.m.s.d. shown as a grey mesh and the *mF*_o_ − *DF*_c_ difference maps contoured at 3.5 r.m.s.d. shown as red (negative) and green (positive) solid surfaces

PDB entry 5s7c is a 1.31 Å resolution structure of human activin receptor type-1 complexed with several ligands determined as part of a fragment-screening study and deposited with 44 other structures in a group deposition. One of the ligands modelled in PDB entry 5s7c is 2-hydroxypyridine, whose electron-density fit is excellent (Fig. 5 ▸*a*), and there are no issues with geometry validation. However, examination of the molecular interactions between the ligand and protein shows that the pyridine-ring N atom, a hydrogen-bond acceptor, points towards a main-chain carbonyl O atom that is also a hydrogen-bond acceptor (Fig. 5 ▸*a*). This interaction between two acceptors would be unfavourable. In aqueous polar environments, 2-hydroxypyridine tautomerizes to 2-pyridone (Forlani *et al.*, 2002 ▸), making it likely that the 2-pyridone tautomer would predominate when bound to a protein (Fig. 5 ▸*b*). Remodelling the ligand to 2-pyridone provides an equally excellent electron-density fit (Fig. 5 ▸*b*). The ligand now forms good hydrogen-bond contacts with the protein. The slight alteration to this ligand does not warrant a redeposition of the PDB entry 5s7c structure. Instead, fragment-screening studies using 2-hydroxypyridine/2-pyridone should initially model this molecule as the pyridone tautomer.

Notably, even for this high-resolution structure, the two tautomers have equivalent fits to the electron density. It is not possible to distinguish between a carbonyl double bond and a hydroxide single bond by examining the fit to electron density. Accordingly, assigning the tautomeric state of a ligand depends on examining its interactions with surrounding groups and detailed knowledge of the compound’s behaviour at different pH values in solution. Often, it is not possible to unambiguously identify the tautomeric state of a ligand, and downstream users of PDB structures should bear this in mind.

### 4.6. Do the deposited coordinate and X-ray dataset files match?

Problems can also occur during the final deposition of the refined model to the PDB or to an internal corporate database. Where possible, the mmCIF files from the program used in the final round of refinement should be used, as these will include map coefficients and ligand restraints. It is vital to ensure that the X-ray structure-factor file corresponding to the coordinates is used in the deposition. This can be checked by downloading the annotated model and reflection files from the PDB, calculating *R* factors and examining the electron-density maps around the ligand of interest.

#### 4.6.1. Case study

**Figure 6:**
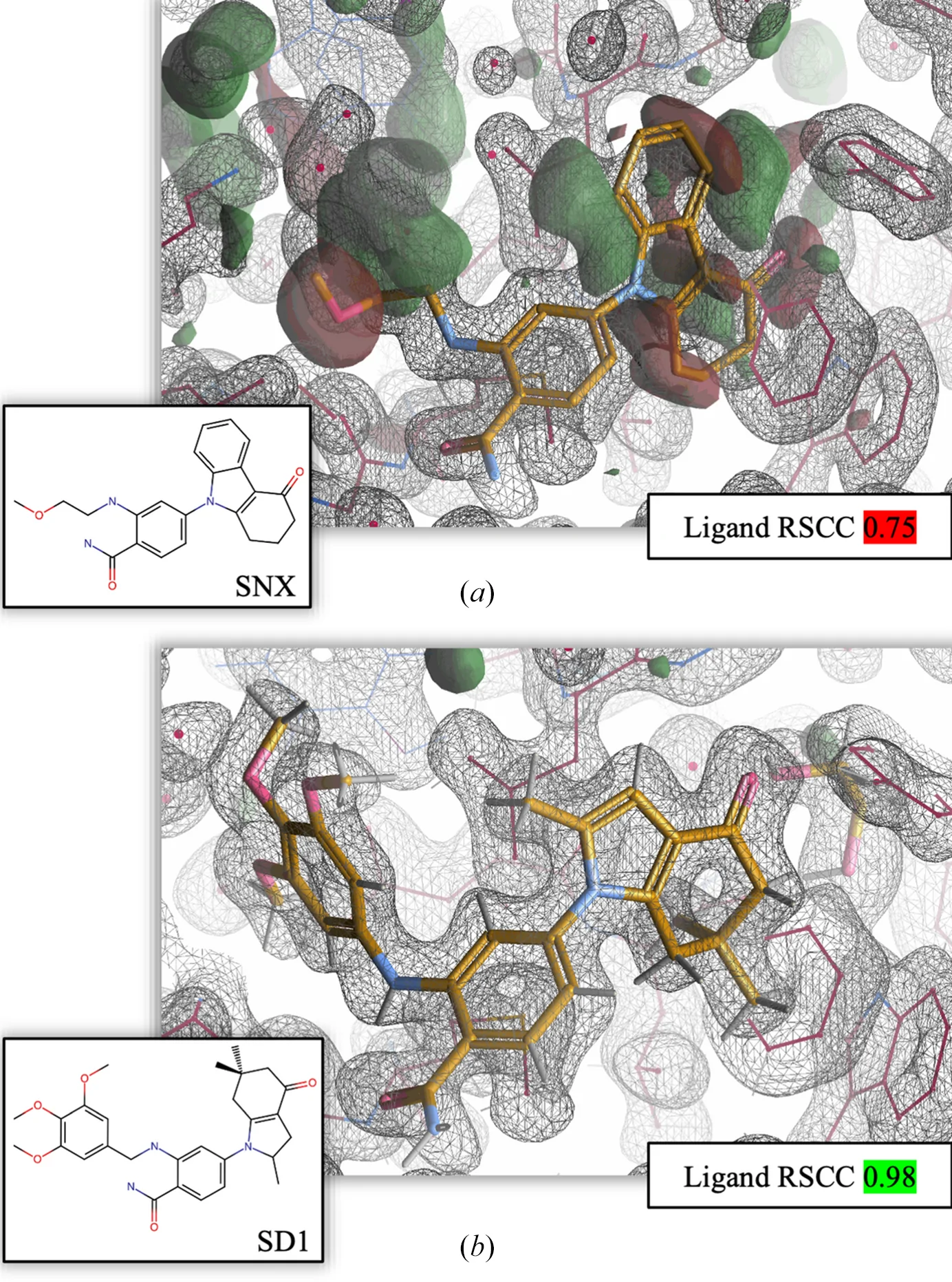
The wrong X-ray dataset was deposited with PDB entry 3d0b. (*a*) Re-refinement of the PDB entry with *BUSTER* produces numerous blobs of difference density and a poor real-space correlation coefficient. (*b*) Reinterpreting the electron-density map indicates that the ligand in the X-ray dataset is in fact SD1. 3D figures produced using *Coot* with the *BUSTER* 2*mF*_o_ − *DF*_c_ map contoured at 1.3 r.m.s.d. shown as a grey mesh and the *mF*_o_ − *DF*_c_ difference maps contoured at 3.5 r.m.s.d. shown as red (negative) and green (positive) solid surfaces. Aligned 2D chemical diagrams were produced using the *Marvin JS* sketch tool (*Marvin* 22.1.1, ChemAxon; https://www.chemaxon.com).

PDB entry 3d0b is a 1.74 Å resolution structure of Hsp90 complexed with a novel benzamide-based inhibitor (Barta *et al.*, 2008 ▸). Re-refinement of the PDB entry (supporting information website Section S6) yields substantial difference density around the inhibitor (Fig. 6 ▸*a*), indicating that the deposited X-ray dataset corresponds to a different inhibitor, as reported by Joosten, Womack *et al.* (2009 ▸). The likely chemical structure of the inhibitor in the PDB entry 3d0b dataset was inferred from careful examination of the binding-site electron density. Searching chemical databases for the inferred inhibitor results in a match to the PDB chemical component SD1, a related benzamide-based inhibitor from PDB entry 3mnr, deposited by the same group two years later (Fadden *et al.*, 2010 ▸). Re-refinement of the PDB entry 3d0b dataset with the SD1 inhibitor yields an excellent fit to the electron density (Fig. 6 ▸*b*). We hypothesize that there was an inadvertent data mismatch, in which an incorrect X-ray dataset was deposited with the PDB entry 3d0b coordinates. It is interesting to note that the X-ray reflection dataset deposited with PDB entry 3d0b is distinct from the PDB entry 3mnr dataset, with slightly different diffraction limits. The collection dates differ by two days. This example underscores the critical need for checks during deposition. If the X-ray dataset used for the PDB entry 3d0b model remains available, it should be deposited in the wwPDB; otherwise, the entry should be obsoleted without replacement as it lacks the correct experimental data. It is regrettable that this problem was not cleared up in 2009, as it was reported by Joosten, Womack *et al.* (2009 ▸).

### 4.7. Is the ligand of interest’s chemical markup correct after deposition?

Once a structure has been deposited, it is important to ensure that the ligand chemistry defined in the database matches that used in the refinement. For PDB entries, ligand chemistry is defined in the PDB chemical component dictionary (Westbrook *et al.*, 2015 ▸). Downstream users of structures, for example the *Boltz* protein–ligand structure and binding affinity prediction tool (Wohlwend *et al.*, 2025 ▸), rely on the PDB chemical component dictionary to obtain ligand chemistry and will encounter problems if the chemical definition for a ligand is not correct. PDB deposition is often performed at the end of a project when the depositors have already moved on to new tasks, but getting details such as these correct is important.

It should be noted that the PDB chemical definition of a compound is usually for a neutral form, and so for charged ligands it can differ from the form used in refinement. So, it is advisable to include explicit ligand H atoms in the coordinates file, as this clarifies the chemistry, including charge state, used during refinement for downstream users of the structure. Including explicit ligand H atoms also makes the tautomeric form clear when examining the coordinates and helps in assessing ligand protein coordinates.

Although the wwPDB accepts restraint dictionaries for ligands at deposition, these are not currently made publicly available when the entry is released. It is regrettable that these metadata are kept private (Terwilliger & Bricogne, 2014 ▸), as they would clarify the ligand chemistry used in refinement and be valuable in ligand validation.

#### 4.7.1. Case study

**Figure 7:**
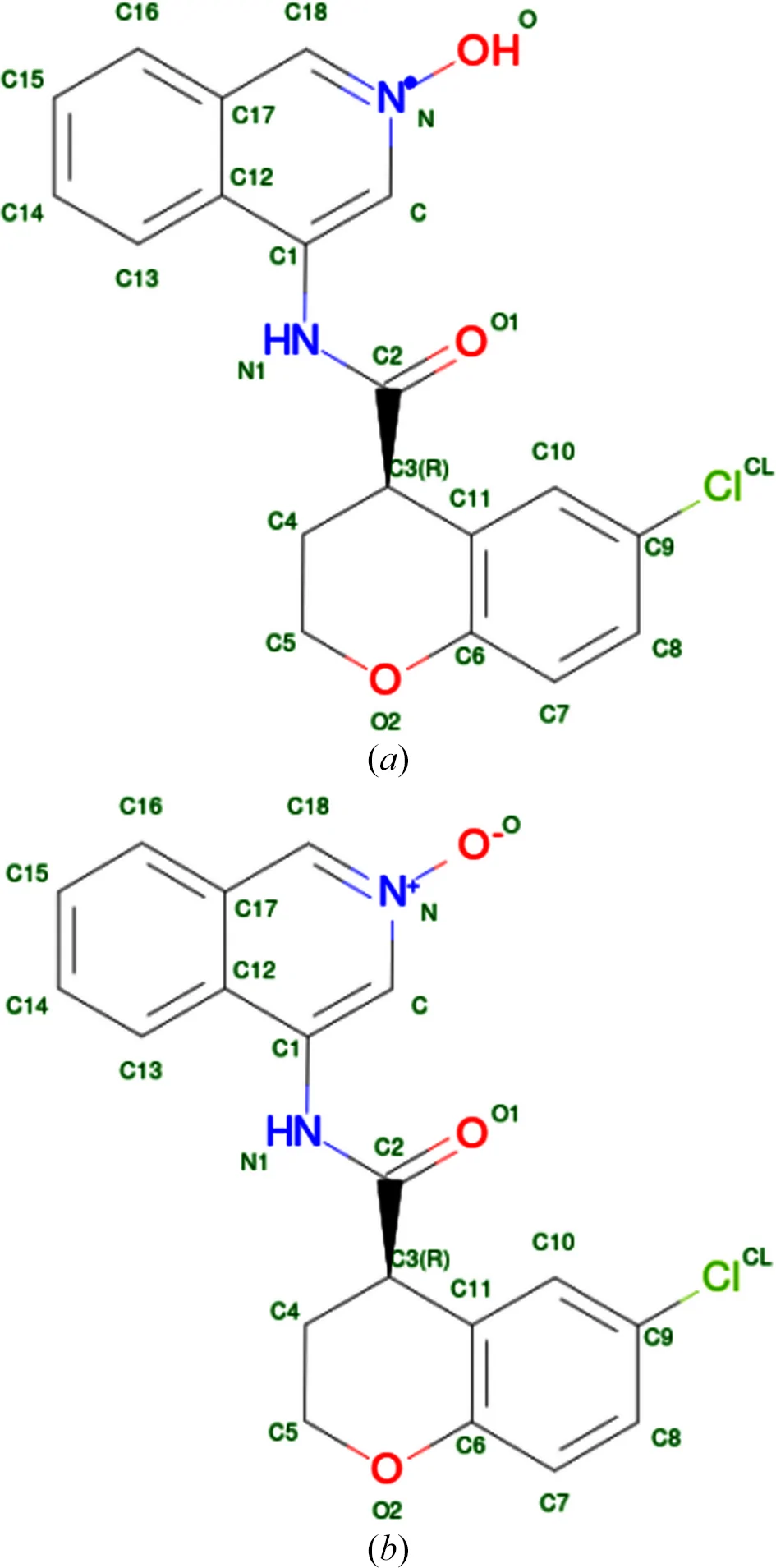
The PDB chemical component definition for PWR needed correction. (*a*) The original definition incorrectly described the chemistry of the isoquinoline-*N*-oxide group, adding an H atom to the O atom and indicating that the N atom is a free radical with a dot. (*b*) The definition after correction. Although the difference is small, it is not trivial, as issues like this will negatively affect the downstream processing of the structure.

PDB chemical component definition PWR was created for PDB entry 7gh0, one of the 367 structures of SARS-CoV-2 inhibitors deposited as part of the COVID Moonshot project (Boby *et al.*, 2023 ▸). In the original definition of the component, the chemical markup for the isoquinoline-*N*-oxide ring was incorrect (Fig. 7 ▸*a*). The component was corrected when we reported the problem to the PDB (Fig. 7 ▸*b*).

## 5. Discussion

As discussed in Section 1, many publications highlight PDB entries with flawed ligand modelling or other issues that could be improved. The case studies presented here, drawn from the PDB, further demonstrate the range of problems that can arise, from fundamental (*e.g.* modelling a ligand in solvent density) to subtle (*e.g.* using the incorrect tautomeric state). The implications of some errors can be severe, as they propagate misleading information about protein–ligand interactions and impede efficient data mining (Dauter *et al.*, 2014 ▸; Wlodawer *et al.*, 2018 ▸).

The PDB entry 3hnb and 8ivl case studies are compelling examples of the importance of considering the null hypothesis. Researchers must be willing to conclude that their ligand did not bind, rather than forcing a molecule into ambiguous density that is better explained by solvent, an endogenous ligand or cryoprotectant. The PDB entry 1udt (Sildenafil) case highlights the danger of flexible six-membered rings, which are easily mis-modelled during fitting and can become ‘stuck’ in high-energy conformations. It is essential to use geometry validation tools, such as *Mogul*, in conjunction with refinement. Similarly, the PDB entry 3tu1 and 5s7c cases show that the chemical ‘ground truth’ (chirality, tautomers) must be accurate for refinement to yield the best results.

PDB entry 3hnb illustrates how a PDB structure with a poorly modelled ligand can confuse subsequent studies. Nicolaes *et al.* (2014 ▸) noted that the ligand modelled in PDB entry 3hnb was problematic because it was positioned in a region with a very flat surface, and three different computational docking methods could not place the ligand. They concluded that ‘additional investigations are therefore needed’. In practice, the ligand of interest in PDB entry 3hnb is not experimentally supported and should have been excluded from the study. Users of protein–ligand co-structures from the PDB should exclude structures with poor real-space correlation coefficients (RSCC) for the ligand and inspect the electron density around the modelled ligand position before using the structure.

For users of the PDB without the necessary crystallographic knowledge, the RCSB website now provides a ‘Ligand Structure Quality Assessment’ slider based on the analysis of RSCC by Shao *et al.* (2022 ▸), which classifies PDB entry 3hnb (Supplementary Fig. S2A-3) and PDB entry 8ivl (Supplementary Fig. S2B-3) as ‘Worse’. However, the slider fails to flag issues in any other PDB ligands examined here (Table 1 ▸).

As part of the work for this article, we have made a detailed comparison between the *Mogul* analyses performed by the wwPDB Validation Report (wwPDB VR), CCDC *Mercury* programs and *Buster-report* (supporting information website Section S7). The comparison highlighted problems in the way *Mogul* is run in the wwPDB VR, leading to erroneous information. There was a problem with the chemical markup for many ligands with aromatic bonds, such as adenine and sildenafil. Incorrect chemical markup led to erroneous reports of outliers and to reports that there were no CSD matches for parts of ligands that are well represented in the CSD. This problem was reported to the wwPDB and has now been fixed (wwPDB, 2026 ▸). In addition, the wwPDB VR uses an inappropriate criterion when presenting *Mogul* ring analysis, resulting in a ligand with a poor ring conformation being classified as ‘not a ring outlier’. For example, the current wwPDB VR for PDB entry 1udt wrongly classifies the sildenafil ligand piperazine ring as ‘not an outlier’ (Supplementary Fig. S7-1). *Buster-report* (Supplementary Fig. S7-2) and *Mercury* (Supplementary Fig. S7-4) both classify the piperazine ring as being problematic and it is in a distorted envelope conformation (Supplementary Fig. S7-3). A similar misclassification is found for a glucoside pyranose in PDB entry 2evs, used as an example of a problematic ring by Liebeschuetz *et al.* (2012 ▸) (Supplementary Fig. S7-5) (which is in a half-chair conformation), but the wwPDB VR classifies it as ‘not an outlier’, in conflict with *Mercury* classification as ‘unusual’ (Supplementary Fig. S7-7). We have reported this problem to the wwPDB, but it has not yet been fixed (wwPDB, 2026 ▸).

The PDB entry 5s7c example shows that considering alternative tautomeric states for ligands is particularly challenging. It would be helpful for restraint dictionary-generation tools to automatically provide dictionaries for alternative tautomers. To identify cases where an alternative tautomer should be modelled, ligand-validation tools need to detect and highlight unfavourable hydrogen-bond interactions (between two hydrogen-bond acceptors or donors). The recently developed *In-Pocket* analysis method (Menezes *et al.*, 2026 ▸) uses a quantum-mechanical representation of the ligand and its binding pocket. It appears to show particular promise for assigning ligand tautomeric and charge states.

### 5.1. What to do about problematic entries in the PDB database

A variety of approaches have been taken by authors when reporting a problematic PDB structure (Table 2 ▸).

It is good practice for articles that point out a problem in a PDB structure to cite the associated publication rather than just stating the PDB accession code, because this makes the information accessible from citation searches. Europe PMC (Ferguson *et al.*, 2021 ▸) now allows users to find articles citing a PDB entry, but it is unlikely that non-structural-biology researchers would use this feature.

Given that analysis of PDB structures is fundamental to modelling and prediction algorithms, structural corrections really should be made available through the PDB. This is preferable to reporting corrections in presentations or in a publication figure, as we have done (Liebeschuetz *et al.*, 2012 ▸; Smart & Bricogne, 2015 ▸), because the improved structure is made available to other scientists. Perhaps journals should consistently require that reports of structure reinterpretations be deposited in the PDB, just as they do for new structures. An alternative is to make the reinterpreted structure available in a publication’s supplementary materials (Dialpuri *et al.*, 2024 ▸), but this makes the improvement inaccessible to most users. The Covid-19.bioreproducibility.org database (Wlodawer *et al.*, 2020 ▸; Brzezinski *et al.*, 2021 ▸) includes more than 100 reinterpreted PDB structures, many of which exhibit significant improvements. Despite the database’s encouragement of the authors of the original depositions to review and redeposit the improved structures, this has not yet been done in most cases.

There are two paths to correct a problematic entry in the PDB. The original depositors may make or approve updates at any time. At one time, any update to a PDB entry resulted in the issuance of a new PDB accession code, which proved an impediment to the deposition of improvements (Terwilliger & Bricogne, 2014 ▸). To address this, the PDB introduced entry versioning in 2017, whereby most updates to PDB entries by the original authors retain the same accession code but use a different version number (wwPDB, 2017 ▸). Our experience contacting depositors and helping with improved replacement entries has been mixed; often emails are ignored, but we have succeeded in arranging updates to several PDB entries, for example to PDB entries 4tzt, 4ckr, 4z9l and 4xxh.

Reinterpretations of PDB structures by third parties can also be deposited in the PDB without the original depositors’ approval, although this process is more involved. These ‘re-refinements’ (that actually normally involve a considerable degree of reinterpretation) require publication in a peer-reviewed journal and are assigned a new PDB accession code (wwPDB, 2025 ▸). This results in the PDB hosting duplicate entries: the original and the new reinterpretation, for the same experimental data. A significant problem, as noted by Wlodawer *et al.* (2018 ▸), is the insufficient cross-referencing *from* the original entry *to* the reinterpretation. Although this is done on the RCSB website, the information is not included in the mmCIF file for the original entry (or on the PDBe website). It is therefore not automatically available to users of the original entry.

We aim to avoid duplication and so will contact the depositors of the original PDB entries, asking them to assist with redepositing updates to the originals (Table 1 ▸), with their cooperation. It is likely that this will prove problematic, as most of the case studies are old, so we will deposit the reinterpreted structures as PDB ‘re-refinement’ entries and record this on the supporting information website.

*PDB-REDO* has pioneered the automated re-refinement and rebuilding of all X-ray structures deposited in the PDB (Joosten, Salzemann *et al.*, 2009 ▸; Joosten *et al.*, 2012 ▸, 2014 ▸). This is a powerful approach that makes improvements routinely available as methods improve. Currently, *PDB-REDO*re-refinement does not refit modelled ligands; instead, it starts from the PDB-deposited coordinates. Automating ligand reinterpretation and improved validation would be highly valuable to users of PDB structures.

## 6. Conclusion

Producing a high-fidelity protein–ligand complex X-ray structure requires constant vigilance and the critical use of a full suite of validation tools. We have outlined a process that encourages crystallographers to pause and validate their work at critical stages. By treating the model as a hypothesis to be challenged, particularly by challenging the ligand’s very presence, we can improve the overall quality of structural data in the PDB and provide a more reliable foundation for biomedical research.

## Supplementary Material

Website providing complete, step-by-step descriptions of the reinterpretations of each PDB entry.: https://gphl.gitlab.io/ligand-pitfalls-paper/

GitLab repositories from which the website is generated.: https://gitlab.com/gphl/ligand-pitfalls-paper/

GitLab repositories from which the website is generated.: https://gitlab.com/gphl/ligand-pitfalls-paper-additional

Permanent archive of repository material.: https://doi.org/10.5281/zenodo.18032110

## Figures and Tables

**Table 1:** Ligand-validation case studies

Pitfall	PDB code	Supporting information website section	Recommendation for PDB entry
A well determined structure with a well modelled ligand of interest	4tzt	S1	Leave unaltered – this is a control good structure
The ligand of interest has been modelled into electron density better explained as water molecules	3hnb	S2A	Obsolete (or deposit the null-hypothesis structure to mark the problem)
The cholesterol ligand of interest has been modelled into electron density better explained by an endogenous fatty acid	8ivl	S2B	Replace 8ivl with the reinterpreted structure with fatty acid bound
Mistake in the chemistry of the ligand of interest	3tu1	S3	Update the entry with reinterpretation
Part of the ligand has a poor geometry and/or fit	1udt	S4	Update the entry with reinterpretation
The ligand of interest has been modelled in the wrong tautomer	5s7c	S5	Leave unaltered
Deposited X-ray data are for a different ligand	3d0b	S6	Redeposit with the correct X-ray data or obsolete the entry
Problem in the chemical component definition for ligand, chemical component PWR	7gh0	n/a	The issue has already been corrected by the wwPDB

**Table 2:** Approaches to reporting a problematic PDB structure

Approach	Example publication using the approach	Can readers of the PDB article find out about the problem?	Can users access the reinterpreted coordinates?	Are improved coordinates available in the PDB?
Cooperate with the original depositors to deposit the improvement	Smart & Bricogne (2015 ▸)	Yes	Yes, easily	Yes, replacing the original
Describe the problem and deposit reinterpreted coordinates with the PDB	Wlodawer *et al.* (2018 ▸)	Yes	Yes, easily	Yes, but alongside the original
Describe the problem and provide the reinterpreted coordinates in supplementary materials	Dialpuri *et al.* (2024 ▸)	Yes	Yes, but with some difficulty	No
Just describe the problem in a figure or the text citing the PDB code and the associated publication	Joosten, Womack *et al.* (2009 ▸)	Yes, through citation search	No	No
Just describe the problem in a figure or the text citing just the PDB code but not the associated publication	Casagrande *et al.* (2025 ▸)	Not easily	No	No
Just describe the problem in a figure or the text but anonymize the structure	Kleywegt (2007 ▸)	No	No	No

## Data Availability

A supporting information website providing complete, step-by-step descriptions of the reinterpretations of each PDB entry is available at https://gphl.gitlab.io/ligand-pitfalls-paper/. The website is generated from the GitLab repositories https://gitlab.com/gphl/ligand-pitfalls-paper and https://gitlab.com/gphl/ligand-pitfalls-paper-additional. The repository material is permanently archived at Zenodo: https://doi.org/10.5281/zenodo.18032110.
